# Ischial screw fixation can prevent cup migration in 3D-printed custom acetabular components for complex hip reconstruction

**DOI:** 10.1186/s42836-022-00154-3

**Published:** 2022-12-06

**Authors:** Suroosh Madanipour, Daniel Lemanu, Chethan Jayadev, Will Aston, James Donaldson, Jonathan Miles, Richard Carrington, Robert McCulloch, John Skinner

**Affiliations:** Joint Reconstruction Unit, Royal National Orthopaedic Hospital, Stanmore, HA7 4LP UK

**Keywords:** Custom acetabular components, Custom acetabulum, Revision, Hip, Arthroplasty, Ischial fixation

## Abstract

**Introduction:**

Custom acetabular components have become an established method of treating massive acetabular bone defects in hip arthroplasty. Complication rates, however, remain high and migration of the cup is still reported. Ischial screw fixation (IF) has been demonstrated to improve mechanical stability for non-custom, revision arthroplasty cup fixation. We hypothesize that ischial fixation through the flange of a custom acetabular component aids in anti-rotational stability and prevention of cup migration.

**Methods:**

Electronic patient records were used to identify a consecutive series of 49 custom implants in 46 patients from 2016 to 2022 in a unit specializing in complex joint reconstruction. IF was defined as a minimum of one screw inserted into the ischium passing through a hole in a flange on the custom cup.

The mean follow-up time was 30 months. IF was used in 36 cups. There was no IF in 13 cups. No difference was found between groups in age (68.9 *vs.* 66.3, *P* = 0.48), BMI (32.3 *vs.* 28.2, *P* = 0.11) or number of consecutively implanted cups (3.2 *vs.* 3.6, *P* = 0.43). Aseptic loosening with massive bone loss was the primary indication for revision. There existed no difference in Paprosky grade between the groups (*P* = 0.1). 14.2% of hips underwent revision and 22.4% had at least one dislocation event.

**Results:**

No ischial fixation was associated with a higher risk of cup migration (6/13 *vs.* 2/36, *X*^2^ = 11.5, *P* = 0.0007). Cup migration was associated with an increased risk for all cause revision (4/8 *vs.* 3/38, *X*^2^ = 9.96, *P* = 0.0016, but not with dislocation (3/8 *vs.* 8/41, *X*^2^ = 1.2, *P* = 0.26).

**Conclusion:**

The results suggest that failure to achieve adequate ischial fixation, with screws passing through the flange of the custom component into the ischium, increases the risk of cup migration, which, in turn, is a risk factor for revision.

**Supplementary Information:**

The online version contains supplementary material available at 10.1186/s42836-022-00154-3.

## Introduction

Massive acetabular bone defects present a significant challenge to the arthroplasty surgeon [1]. Multiple techniques have been described to manage this problem, including impaction grafting, jumbo uncemented cups with or without porous metal augments and non-customized cup cage and customized cage constructs. Primary fixation and initial stability of the construct are the key to success for all techniques.

Custom acetabular components have become an established method of treating massive acetabular bone defects, with modern designs including porous metal technology to facilitate bone ingrowth and therefore further ensure stability [2]. Custom acetabular reconstructions provide good mid-term results with a 80% survivorship at 5 years [3, 4]. Furthermore, custom constructs can help restore the hip center and achieve a reliable fit in complex defects, which can confer further stability compared to ‘off-the-shelf’ constructs [5]. Much of the published literature on custom acetabular reconstruction is related to Paprosky 3B type defects. However, due to the growing evidence of reproducible results, the indications for their usage has broadened to include multiple revised hip replacements and lesser acetabular defects.

However, complication rates remain high [6] and amongst these, migration of the construct can present a challenging issue. Taunton *et al*. described cup migration in 16% of their case series [7], whilst Barlow *et al*. reported radiographic loosening and ischial flange pull-off in 21% of their cases [8]. A tilt in the construct will result in a change in the cup inclination, which can potentially result in instability. Most commonly, the cup 'abducts', thereby increasing the cantilever forces upon the superior fixation and risking failure of the construct [9]. Ischial fixation has been demonstrated to help resist this 'abduction' force upon the implanted cup [10].

To this date, there is little available research evaluating the impact of ischial screw fixation in custom acetabular components. Ischial screw fixation (IF) has been demonstrated to improve mechanical stability for non-custom, revision arthroplasty cup fixation in laboratory based biomechanical studies, and prevent component migration in clinical studies [11, 12]. Jones *et al.* have previously reported on factors that influence custom implant survival and found ischial fixation to be associated with increased longevity [10].

This study therefore aimed to evaluate implant survivorship in our institution series of custom acetabular reconstructions and the role of ischial screw fixation through the inferior flange in aiding construct stability, and its effect on cup migration.

## Methods

From electronic patient records, 49 custom acetabular components were identified in 46 consecutive patients from 2016 to 2022 in a tertiary orthopedic unit specializing in complex joint reconstruction (Table 1). This represents 5% of the unit's revision workload, with 840 revision hip procedures performed during the time of the study period.

**Table 1:** Patient characteristics

	Non-ischial fixation group		Ischial fixation group			Total	
Implants	13		36			49	
Age (Mean)	68.9 (SD 6.8)		66.3 (SD 12.6)		*P* = 0.48	67	
Total no. of implanted cups	3.2 (SD 1.0)		3.6 (SD 1.5)		*P* = 0.43	3.5	
Sex (No. of females)	11/13 (85%)		28/36 (78%)		*P* = 0.6	39/49 (80%)	
BMI	32.3 (SD 10.1)		28.2 (SD 6.5)		*P* = 0.11	29.3	
Paprosky Grade	3A	2	2B	3	*P* = 0.10	2B	3
	3B	10	2C	2		2C	2
	N/A	1	3A	16		3A	18
			3B	15		3B	25
						N/A	1

Implants were manufactured by Lima (Villanova di San Daniele di Friuile, Udine, Italy), Materialise (Leuven, Belgium) and Adler Ortho (Cormano, Italy). Procedures were all carried out by revision arthroplasty specialists.

**Fig. 1:**
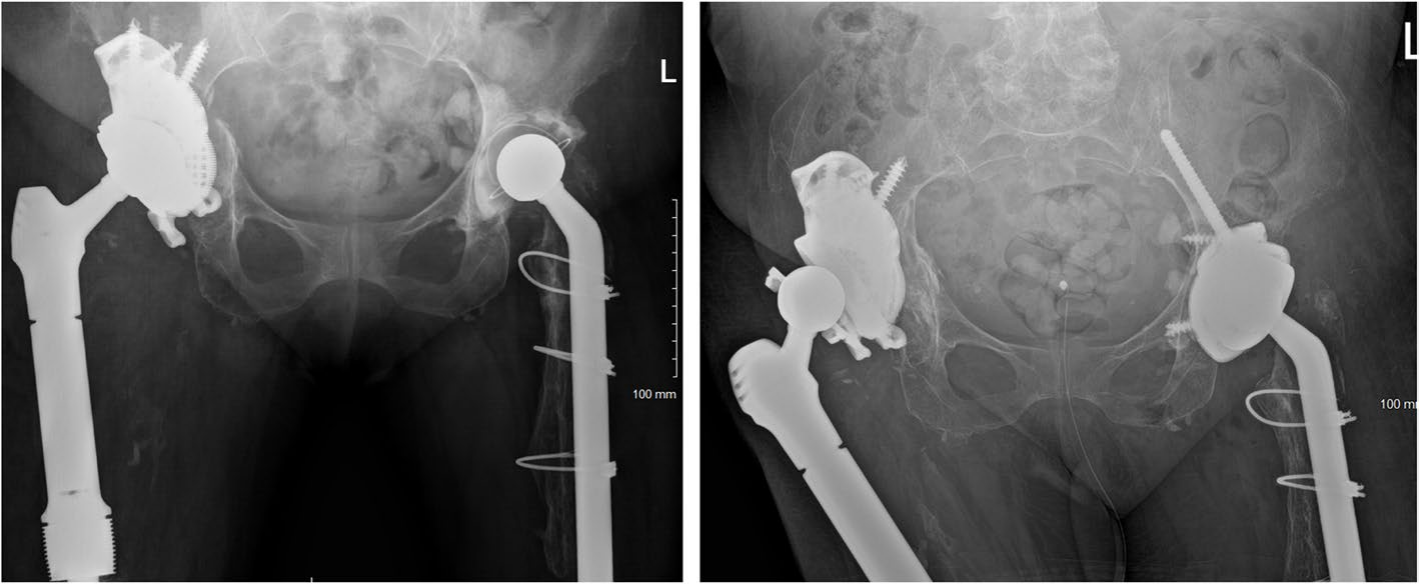
A patient with bilateral custom acetabular components and ischial fixation at 5 year (right hip) and 4 year (left hip) follow up

**Fig. 2:**
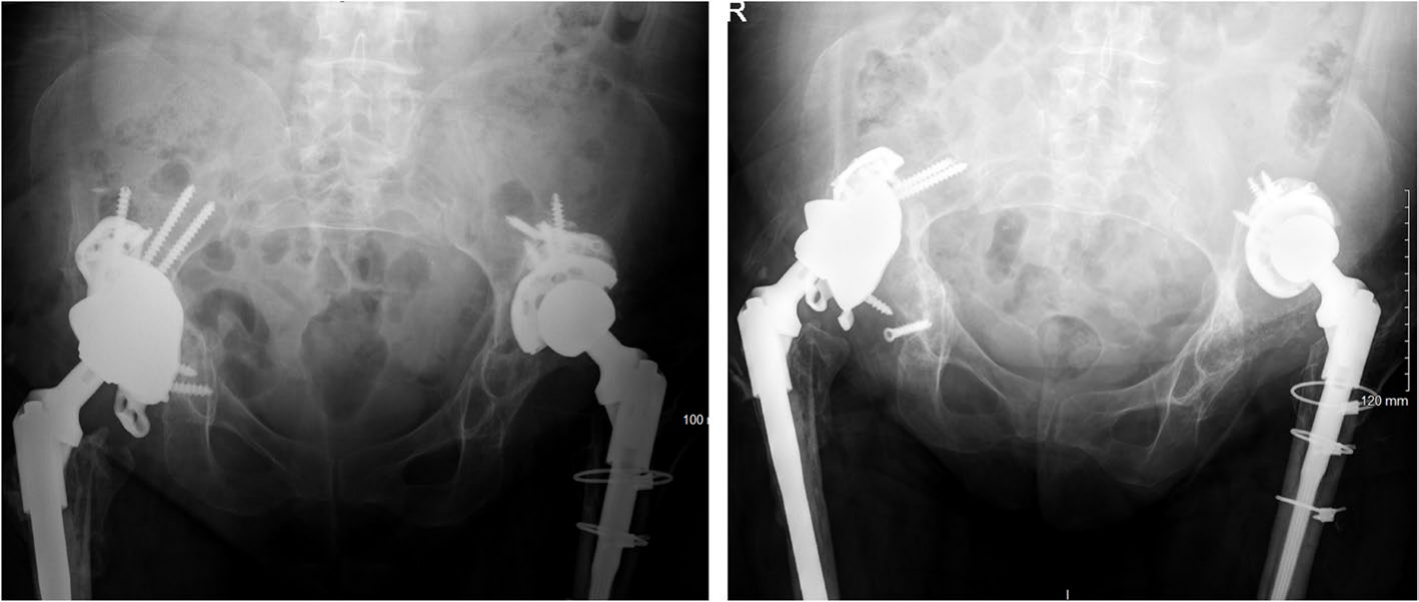
Custom acetabular component without ischial fixation (left image) with subsequent cup migration and reoperation for instability (right image)

**Fig. 3:**
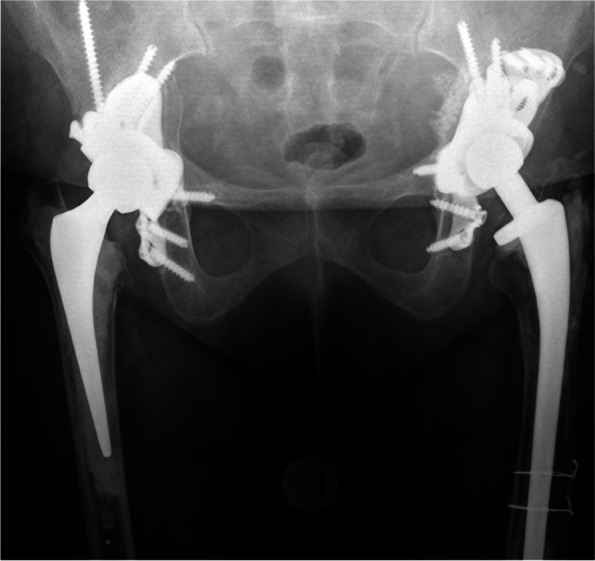
Custom acetabular component without ischial fixation (left image) with subsequent cup migration. The ischial flange was not utilized in the initial fixation

Patients were categorized into either having ischial fixation (IF) (Fig. 1) or receiving no ischial fixation (no-IF) (Figs. 2 and 3), where IF was defined as a minimum of one screw inserted into the ischium passing through a hole in a flange on the custom cup. Selection criteria for IF were based on surgeon preference.

Demographic data, including age at implantation, BMI and sex, were recorded using anaesthetic charts and patient notes. Following review of operation notes, clinic letters and imaging, the following data were recorded: the number of consecutively implanted cups including the current custom component, the indication for surgery, the date of implantation and Paprosky grade. Concurrent femoral implant revision, the use of ischial or superior pubic ramus fixation and the number of ischial screws were noted. The primary outcome measures were postoperative cup migration, dislocation and revision. Cup migration was measured by comparing immediate postoperative radiographs and/or computed tomography with images at the final follow-up to calculate change in cup inclination and change in distance of the dome of the cup from the true floor of the acetabulum using the picture archiving system (McKesson, Irving, TX, USA). Migration was defined as at least 10-degree change in cup inclination and/or 1-cm displacement from the true floor of the acetabulum. This radiographic assessment was undertaken by a senior resident (SM) and verified by an arthroplasty fellow (DL) and a revision arthroplasty consultant (RM) who were not involved in the management of the cases. Verification was therefore blinded with respect to operating surgeon and was not performed by any operating surgeon. Revision was defined as reoperation requiring component (including liner) exchange. Reoperation was defined as any return to theatre.

All patients that had at least one postoperative and one follow-up radiograph were included. Cup migration was deemed to be an early complication and thus there was no minimum follow-up period if this requirement was met. One patient with extensive hemipelvic resection and no residual ischium was removed and one patient who died secondary to cement reaction during femoral revision was excluded.

Within our institution, all revision arthroplasty cases are discussed within a multi-disciplinary setting and any cases that may potentially require a customized reconstruction are scrutinized along with an engineering plan. All custom designs used within our series were based upon metal artefact reduction CT scans of the whole pelvis, allowing for assessment of the center of rotation for the failed hip replacement. Additive manufacturing methods were used for all implants within the series with areas of porous titanium to allow for host bone integration to the construct.

Intraoperatively, all procedures utilized an extended posterolateral approach. Soft tissue dissection around the ischium was performed to visualize this area. 3D-printed models of the custom models were used to aid with preoperative planning and intraoperatively to guide whether the acetabulum had been sufficiently prepared to accommodate the custom prosthesis. Screws were all placed through pre-designed screw holes and were either cortical or cancellous screws with no locking constructs. No screw adjuvants were used such as cement augmentation or bone substitutes.

Statistical analysis was performed using *t*-test and chi-square test for continuous and categorical variables respectively. Fischer exact test was used when sample sizes were smaller. Continuous data are reported as a mean with a standard deviation. Statistical significance was determined as *P* < 0.05.

## Results

Forty-nine custom implants were used in 46 patients (Table 2). The mean follow-up time was 30 months. Ischial fixation was used in 36 cups. There was no ischial fixation in 13 cups. No difference was found between groups in age (68.9 *vs.* 66.3, *P* = 0.48), BMI (32.3 *vs.* 28.2, *P* = 0.11) or number of consecutively implanted cups (3.2 *vs.* 3.6, *P* = 0.43). Aseptic loosening with massive acetabular bone loss was the indication for revision in the majority of cases. This indication was distributed evenly between groups (*P* = 0.97). Infection was the second most common indication, with no difference between groups. (Fischer exact test value 1, *P* > 0.05). There was no difference in Paprosky grade between the groups (*P* = 0.1). One case in the non-IF group underwent primary resection for chondrosarcoma and was not graded according to Paprosky. Overall, 14.2% of hips underwent revision and 22.4% had at least one dislocation event, and 16.3% of cups migrated. Each cup migration occurred with increased cup inclination/abduction and lateral translation from the true floor of the acetabulum.

**Table 2:** Outcomes

	Non-ischial Fixation Group	Ischial fixation group		Total
Implants	13	36		49
Cup Migration	6/13	2/36	*P* = 0.0007	
Revision – All Cause	3/13 (23.1%)	4/36 (11.1%)	*P* = 0.29	7/49 (14.2%)
Dislocation	2/13 (15.3%)	9/36 (25%)	*P* = 0.48	11/49 (22.4%)
Non-revision Reoperation	1/13	2/36		3/49
Sciatic Nerve Injury	1/13			1/49
Femoral Nerve Injury		1/36		1/49
Prosthetic Joint Infection	2/13	2/36		4/49
Deaths	4/13	4/36	*P* = 0.10	8/49

No ischial fixation was associated with a higher risk of cup migration (6/13 *vs.* 2/36, *X*^2^=11.5, *P* = 0.0007). Cup migration was associated with an increased risk for all-cause revision (4/8 *vs.* 3/38, *X*^2^=9.96, *P* = 0.0016, but not dislocation (3/8 *vs.* 8/41, *X*^2^=1.2, *P* = 0.26) (Table 3). There was no difference between groups when comparing the number of hips undergoing at least one dislocation (2/13 *vs.* 9/36, *X*^2^=1.99, *P* = 0.48). There was no difference in all-cause revision between groups (3/13 *vs.* 4/36, *P* = 0.29).

**Table 3:** Cup migration association with dislocation or revision

	Cup Migration	No Cup Migration	
Revision	4/8	3/41	*P* = 0.0016
Dislocation	3/8	8/41	*P* = 0.26

Within the non-IF group, six out of thirteen cups underwent migration with a mean of 29 degrees of increased abduction/inclination and 24-mm translation from postoperative radiographs to the final follow-up films. Of these, 2 cups were revised to a second custom-made acetabular component, one of which was implanted without ischial fixation and migrated again but was not subsequently revised. The other was revised to a custom cup with ischial fixation and did not migrate at 16 months of follow-up.

Within the IF group, two out of thirty-six cups underwent migration. One migrated 34 mm from the acetabular floor and had an increase of over 40 degrees in inclination angle within 2 months of implantation. Pre- and postoperative CT revealed significant osteolysis within the ischium and subsequently ineffectual ischial fixation screws. A second patient in this group developed migration of the cup 11 mm from the acetabular floor and 9 degrees of increased cup inclination within 13 months of implantation, but suffered from proximal femoral periprosthetic fracture and dislocation with revision to a proximal femoral replacement and custom acetabulum retained. Within the IF group, one cup had 4 screws fixed into the ischium, 11 cups had 3 screws, 17 had 2 screws and 7 had one screw. Those cups that had 3 or more ischial fixation screws underwent no cup migration, whilst there were 2 migrations in the 24 cups which had 1 or 2 ischial screw(s) inserted. This effect, however, was not statistically significant. (Fishers exact test, *P* = 0.5429).

Within the IF group, one Lima ProMade and one Materialise aMace cup underwent migration. Within the non-IF group, two Adler Ortho, three Lima ProMade and one Materialise aMace cup developed migration. This distribution showed no statistical significance.

Overall, there were four cases of prosthetic joint infection, with two in each group. Three were treated with long-term suppressive antibiotics and one received a Girdlestone procedure. There was one case of sciatic nerve palsy in the non-IF group and one case of femoral nerve palsy in the IF group.

## Discussion

The management of complex acetabular defects is challenging. Nonetheless, the concepts of restoration of the hip center and biomechanics, reconstruction of bone defects and stable, robust fixation still apply. Custom designs are a reliable method of achieving the aforementioned technical goals.

Our study identified IF as an important step during revision arthroplasty using custom acetabular reconstruction to prevent cup migration and improve implant survivorship. This finding is in support of current literature which showed higher failure rates with less ischial fixation options [10]. Ischial fixation is not routine in primary or revision hip surgery despite biomechanical benefits. In order to safely pass ischial screws, dissection of the body of the ischium is required, which can increase operative time, blood loss and pose a danger of sciatic nerve injury. This could deter the revision arthroplasty surgeon from doing so. However, in our series there was no increased incidence of foot drop or perioperative mortality in the ischial fixation group.

Custom constructs require collaborative input from engineers and surgeons to optimize shape and design whilst allowing for addressing the practical difficulties encountered during surgical implantation. Initial designs of custom triflange reconstruction relied on an obturator hook or and ischial flange inserted into the bone [5], which provided good survivorship in a complex patient group with revision rates of approximately 30% at 4 years postoperatively [13]. The advent of additive manufacturing methods have allowed for more complex shapes to be reconstructed, enabling more contact of the implant with the host bone and contouring of flanges which allow for precise screw insertion.

There is some evidence that locking screws in the ischium may confer a further advantage in increasing pull-out strength [10, 14]. The use of bone substitute agents such as calcium phosphate when there is ischial bone deficiency may further aid robust ischial fixation [10].

The advent of robotics has ushered in the next era of technological advancement in the field of arthroplasty with its optimal role for surgeons yet to be established. Whilst robot-assisted surgery is being widely integrated into the armamentarium of arthroplasty surgeons globally on the back of ongoing clinical research in primary arthroplasty [15, 16], there is an opportunity for ongoing research evaluating the role of robotics in complex revision arthroplasty where custom components are made to fit in conjunction with hyper-accurate acetabular preparation allowed for by robotics. In our institution, custom constructs are not used routinely for acetabular reconstruction, making up approximately 5% of all revision procedures. Further research in this area has the potential to improve accurate restoration of hip anatomy and biomechanics, improve safety and outcomes, and further increase the reach and utility of the robotic and custom implant technology.

There are limitations to our study. Firstly, this is a heterogenous group in terms of case mix and implant manufacturer. However, both IF and non-IF groups in our series had no significant differences in their demographic variables and therefore this adds to the applicability of our findings. Secondly, the numbers in our study were small, but, in the context of custom acetabular reconstruction, our series size was comparable to the published literature. Finally, there was no control group to allow for comparison with alternative fixation techniques and we did not include functional outcome measures.

## Conclusion

**Fig. 4.**
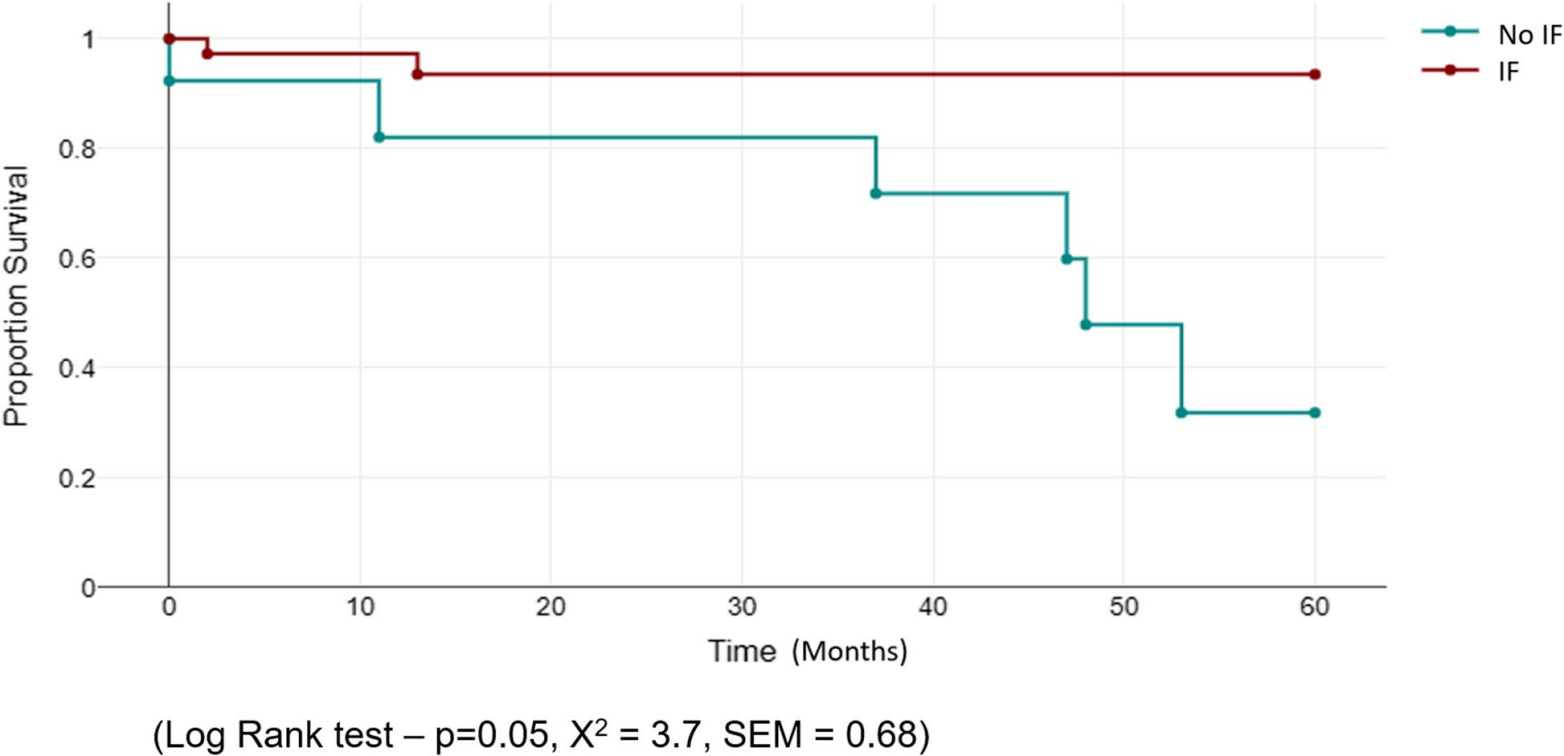
Kaplan-Meier survival curve for ischial fixation (IF) *vs*. non-ischial fixation. (Log Rank test: *P* = 0.05, *X*^2^ = 3.7, SEM = 0.68)

The use of ischial screw fixation when undertaking custom acetabular reconstruction decreases the risk of cup tilt. Patients with construct migration have an increased risk of revision. The authors would recommend ischial screw fixation in all custom implants whenever feasible (Fig. 4).

## Supplementary Information

**Additional file 1.**

## Data Availability

The datasets used and/or analysed during the current study are available from the corresponding author on reasonable request.

## Abbreviation

IF: Ischial Fixation
