# Supplementary material for: Ischial screw fixation can prevent cup migration in 3D-printed custom acetabular components for complex hip reconstruction
Source: Arthroplasty. 2022 Dec 6;4:52. doi: 10.1186/s42836-022-00154-3 (PMC9724270; doi:10.1186/s42836-022-00154-3)
Supplement: Supplementary file 1 — Additional file 1. [file 42836_2022_154_MOESM1_ESM.pdf]

Royal National Orthopaedic Hospital NHS
NHS Trust

Research and Innovation Centre
RNOH, Brockley Hill
Stanmore HA7 4LP
Tel: 020 8909 5825
E-mail: rnoh.research@nhs.net

RNOH Research and Innovation Centre - Confirmation of Service Evaluation

To: Suroosh Madanipour, Rob McCulloch, Daniel Lemanu, John Skinner, James Donaldson, Jonathan Miles, Will Aston, Richard Carrington, Chethan Jeyadev.

Date: 12/08/2022

Project Title: Ischial screw fixation can prevent cup migration in 3D printed custom acetabular components for complex hip reconstruction.

Reg No: SE22.25

The Project Evaluation form you have submitted has been reviewed and based on the HRA “Defining Research” leaflet it was concluded that fits into the category of service evaluation and therefore does not require approval from a Research Ethics Committee (REC).

Service evaluation projects do not require the same level of governance as research of an interventional nature. For the latter, applicable clinical research legislation needs to be considered as part of research set up and conduct. However, any service evaluation projects and those involved must be aware of and follow any relevant guidance and frameworks to ensure patient safety and confidentiality. They must also comply with the clinical governance of the Trust.

Yours sincerely

PP- Vandana

Ufedo Miachi

Research Management and Governance Lead

Research and Innovation Centre
